# Work-related support needs of registered nurses in a neonatal intensive care unit in the Tshwane District

**DOI:** 10.4102/hsag.v28i0.1764

**Published:** 2023-01-12

**Authors:** Funzani Nefale, Nombulelo V. Sepeng, Roinah Ngunyulu

**Affiliations:** 1 Department of Nursing, Faculty of Health Sciences, University of Pretoria, Tshwane, South Africa; 2 Department of Nursing, Faculty of Health Sciences, University of Johannesburg, Johannesburg, South Africa

**Keywords:** neonatal intensive care, registered nurses, work-related support needs, Tshwane District, South Africa

## Abstract

**Background:**

Registered nurses in neonatal intensive care units (NICU) are working under stressful environment caused by the need and commitment to provide care for the critically ill neonates. Therefore, there is an imperative need to know and understand the work-related support strategies that can be adapted for registered nurses working in a NICU in the Tshwane District to enable them to provide quality care for the admitted neonates.

**Aim:**

To explore and describe the work-related support needs of registered nurses working in a specific NICU situated in the Tshwane District.

**Setting:**

The study was conducted in a selected NICU in Tshwane District.

**Method:**

A qualitative, exploratory, descriptive, and contextual design was used in this study. In-depth unstructured individual face-to-face interviews were conducted with nine registered nurses working at the selected NICU of an academic hospital. Thematic data analysis was conducted.

**Results:**

Three themes, namely teamwork between registered nurses and doctors, staff development in the form of peer seminars, workshops and in-service training, and availability of adequate resources within the workplace arose.

**Conclusion:**

This study revealed that the registered nurses working in the NICU in the Tshwane District are in need of work-related support, as it will improve their well-being.

**Contribution:**

The contribution of this study will be used by the hospital management to plan strategies that can be adapted for the betterment of the work environment for registered nurses in the NICU and the hospital in general.

## Background

The intensive care unit (ICU) is regarded as one of the utmost pivotal components within the health system. This is because the patients admitted to these units require advanced technological equipment and medical professionals with advanced skills to manage patients’ conditions (McElroy et al. 2015:629). The neonatal intensive care units (NICU) are established to care for and save neonates who are at risk of losing their life (Matlakala & Botha 2016:49). Despite this, the registered nurses working in NICU are faced with the pressure of providing complex care management needed by the neonates. Towards the end of 2019, the whole world was hit by the coronavirus disease 2019 (COVID-19), and this affected the hospital system negatively. Given the uncertainty, the pandemic added a lot of stress among nurses, including those who were working in NICU (Green et al. 2020:33). Some nurses working in the NICU were infected by COVID-19 and had to be in isolation for 10 days, while others lost their lives which resulted in gross staff shortages. According to Mdzinwa, Voigt and Paruk (2021:1093), there were 2.7 cases per 1000 of severe acute respiratory syndrome coronavirus 2 (SARS‑CoV‑2) among nursing staff. The study was conducted in 2020 from 01 June to 31 August in an academic hospital in the Tshwane District.

According to World Health Organization (WHO 2018:2), an estimated 15 million babies are born prematurely in a year. Approximately 84, 000 of these births happen in South Africa (SA), and even though the bulk of the preterm births take place in hospitals, 10% do not survive (WHO 2018:2). The high number of admitted premature infants contributes to greater professional responsibility and accountability for registered nurses and the demands which, consequently, affect their well-being (Bluebond-Langner et al. 2017:283).

Rogowski et al. (2013:444) explained that in the United States of America (USA), there is understaffing by a tenth of a nurse (per infant) in NICU, and this is associated with 40% higher odds of neonates diagnosed with an infection caused by compromised healthcare. However, in SA, infant mortality is linked to staff shortages, inadequate training, as well as the insufficient number of healthcare facilities (Mabaso, Ndaba & Mkhize-Kwitshana 2014:44). Provenzi, Broso and Montirosso (2018:42) also maintained that registered nurses working in NICU are faced with challenges of caring for critically ill fragile infants who are more likely to have complications such as failure to thrive, jaundice, difficulty in breathing, and dying.

Dealing with these challenges of patient care and professional ideals contributes to workplace stress and as such, there is a need for the provision of work-related support to professional nurses working in NICU. Registered nurses’ work-related support needs differ and depend on what they are experiencing at that particular time. In support, a study conducted in the USA by Smith, Rogowski and Lake (2020:1940) indicated that nurse managers should give nurses work-related support to improve nursing care of new-born neonates and their health outcomes. Grover et al. (2015:179) asserted that the support needs of NICU nurses in the USA can be of emotional, physical and social nature. According to Bindon (2017:110), the work-related support needs for nurses in the USA include staff training, employee benefits, and career advancement.

Workplace stress has an impact on professional nurses across the globe. According to Ayed et al. (2014:100), Palestinian nurses’ physical and mental health is affected by work-related stress. This indicates that they should continue to get administrative support as well as proper training programmes to deal with potentially stressful situations. Support groups, as well as processes consulting with nurse managers have proved successful in resolving issues within interdisciplinary teams in Pakistan (Ayed et al. 2014:100). The support intervention measures such as increased training in identifying and managing work-related stress through assertiveness and relaxation have improved nurses’ job performance in SA (Khamisa et al. 2015:652). Despite this, there is limited information from the literature that describes the work-related support needs of registered nurses working in NICU in SA.

### Problem statement

Registered nurses working in NICU are faced with major challenges in terms of role expectations, patient care and professional ideals, which contribute to workplace stress (Khamisa et al. 2015:652). Most NICU nurses experience psychological burnout, job dissatisfaction, turnover, decreased morale, and fatigue (Fiske 2018:276; Steyn, Myburgh & Poggenpoel 2017:2). The necessity for specialised education and training to manage critically ill neonates, expertise in working with new technologies, and dealing with death are all well-documented stressors in the NICU (Fiske 2018:276). Gutierrez, Candela and Carver (2012:1601) concluded that nurses working in the NICU and receiving high-level support from their managers and co-workers are productive, retained by organisations, and have low-level job stress and absenteeism. The support needs should be identified by those in need, instead of managers and other stakeholders identifying the type of support needed (Bai 2016:27). Despite the research conducted in other countries, there is limited research exploring and describing the work-related support needs of registered nurses working in NICU in SA. This information must be gathered to assist with the development of strategies that can be used by NICU managers to address the support needs of NICU nurses.

## Research methodology

### Design

Qualitative, exploratory, descriptive and contextual designs using thematic analysis were used to explore and describe the work-related support needs of registered nurses working in a specific NICU situated in the Tshwane District of the Gauteng Province (Grove et al. 2012:694).

### Context

The study was conducted at a selected NICU situated in a public academic hospital in the Tshwane District of the Gauteng Province. This NICU has 38 beds, of which 10 beds were used for neonates requiring ICU care and 28 for neonates requiring high care. The neonates who are admitted to this NICU have congenital abnormalities, prematurity, asphyxia, hyaline membrane diseases, neonatal jaundice, and other chronic disorders including immune-compromised diseases such as Human Immune Deficiency, respiratory distress syndrome and diabetes. One of the authors used to work in the selected NICU and observed numerous stressors experienced by the nurses which motivated this study.

### Study population and sampling strategy

The study population included registered nurses working in the NICU, in the Tshwane District of the Gauteng Province. A non-probability purposive sampling technique, as described by Polit and Beck (2014:738), was used to select registered nurses working in the NICU. The NICU at the selected public academic hospital had 12 registered nurses at the time of data collection, and they were recruited with the help of management. The inclusion criteria of this study targeted permanent registered nurses who have registered with the South African Nursing Council (SANC). The nurses who gave consent and had worked for at least 1 year and above at the selected NICU regardless of their gender (*N* = 12; *n* = 9) were included in the study. Therefore, of the 12 registered nurses, only nine met the inclusion criteria for the study and all agreed to participate, and data collection was done among the nine professional nurses. Data saturation was reached with the sixth participant, and three more participants were interviewed to confirm the data saturation.

### Data collection

To avoid coercion, data were collected by an independent researcher who does not have any relationship with the participants. The independent researcher holds a master’s degree in Nursing and has experience in conducting qualitative research. Data were collected in a private and quiet hospital boardroom that was organised by the researcher. The independent researcher adhered to the principles of preparatory and interview phases (De Vos et al. 2011:325). The preparatory phase is the step whereby an independent researcher starts with negotiating an entry phase, to begin with the interviews and establishment of rapport with the participants (De Vos et al. 2011:325). Also, the preparatory phase allows the independent researcher to maintain relationship with the gatekeepers (De Vos et al. 2011:325). In this study, the independent researcher maintained the relationship with the gatekeepers and participants by respecting and adhering to the agreed-upon data collection schedules. During recruitment, the independent researcher informed the participants about the study, and the details included the explanation of the aim of the study, the objectives and the significance of the study. The assurance of the ethical principles was also discussed; all these were done to establish rapport and to ensure that the participants are informed of their right to participate. The independent researcher and the participants adhered to the COVID-19 regulations, i.e. sanitising their hands and surfaces, maintaining social distancing during data collection and always wearing masks when in contact with others.

The interview phase followed when the independent researcher had completed the negotiation phase. Face-to-face unstructured individual interviews were conducted (Brink, Van der Walt & Van Rensburg 2012:158). The independent researcher asked the registered nurses an open-ended question: ‘What are the work-related support needs you would like to receive in the NICU?’ The independent researcher further used probing questions to prompt the participant’s response to the questions asked. The probing questions included:

[*T*]ell me more about the work-related support needs that you have just indicated, you have said you need this type of work-related support, please explain further, why you think you need that specific type of work-related support.

Probing questions were used to seek clarity from their initial response or to encouraged the participants to give more information regarding the subject under study (Brink et al. 2012:158).

Data saturation was reached after the independent researcher had engaged with participant number six when no new information was obtained from the participants. However, the team agreed to continue further and three additional participants were interviewed to ensure that, indeed, there is no new information that might be established. The interviews were recorded with an audio recorder and field notes were taken by the independent researcher to capture the correct information about what was said by the participants during the interviews.

### Data analysis

All the interviews were transcribed verbatim by the researcher. The researcher and the independent co-coder experienced in the field of qualitative research followed the steps of thematic analysis (Braun & Clarke 2013:121). In the first step, the researcher immersed herself and familiarised herself to become acquainted with data by reading it repeatedly. Additionally, by listening to the audio-recorded data that the interviewer collected during interviews and noting any preliminary analytic observations. The same transcripts were sent to the co-coder, to begin with data analysis. In step 2: coding entailed labelling the important features of the data that were relevant to the research question. The researcher and the co-coder coded each data item independently and completed this phase by compiling all of the codes and extracting relevant data. In step 3: searching for themes was done by compiling all the coded data relevant to each theme; the researcher and the co-coder reduced data into small and manageable sets.

In step 4: review of the themes was done; the researcher and the co-coder went through the themes independently, making sure they were relevant to both the coded extracts and the entire data set. In step 5: defining and naming of the theme was done by thoroughly researching and writing the themes by the researcher and the co-coder. The essence of each theme was identified, and a concise, informative name for each theme was created. Step 6: writing up the results entailed weaving the analytic narrative, and data extracts and contextualising it using existing literature (Braun & Clarke 2013:121). The researcher and the co-coder analysed data independently, and thereafter, they had a meeting with the researcher’s supervisors to reach an agreement on the themes and sub-themes of the analysed data before the write-up. This was done to ensure that the results presented in this study were a true reflection of what has been said by the participants while answering the research question of the study.

### Trustworthiness

Lincoln and Guba’s (1986:172–173) methods of credibility, dependability, confirmability, transferability and authenticity were used to ensure trustworthiness. In this study, credibility was demonstrated by allowing the participants to ask questions for clarity. Building a trusting relationship with participants during the recruitment process was done by adhering to appointments and the time scheduled for interviews. Also, trustworthiness was established by member scrutiny between the researchers and the co-coder. This was done by having meetings to reach a consensus regarding the themes and sub-themes of the study which helped to eliminate mistakes and ensured that the findings are supported by participants’ quotations. Furthermore, dependability was ensured by selecting the research method and methodological applications that were appropriate. To enhance confirmability in this study, the interviewer used a voice recorder, transcribed the data verbatim and coded it with the assistance of a co-coder. Transferability was ensured by describing the research design and method in-depth. Authenticity was ensured by confirming that the findings gathered in this study only convey the participants’ views.

### Ethical considerations

Ethical clearance to conduct this study was obtained from the Faculty of Health Sciences Research Ethics Committee of the University of Pretoria (reference number: 883/2019). Prior to data collection, the study received permission from all the relevant stakeholders which included the Provincial Department of Health and the hospital (research setting). Written voluntary informed consent was obtained from all the participants before starting with the data collection. The participants were told that they have the right to halt their participation without any penalty being held against them. The participants were given codes during data collection to ensure anonymity and confidentiality. The researcher deleted the information from the tape recorder after transcribing the data. The transcribed data are kept under a lock-in a-key cupboard, and it will be kept for five years according to the university policy.

## Results

### Demographic characteristics of participants

Nine (*n* = 9) registered female nurses working at the selected NICU agreed to participate in the face-to-face unstructured individual interviews. Two (*n* = 2) of the registered nurses had no clinical specialisation in nursing, five (*n* = 5) of the participants had a specialty in child nursing science, one (*n* = 1) registered nurse had a specialty in intensive care, and the last one (*n* = 1) had a specialty in neonatal nursing.

The themes and sub-themes that were developed from the data collection of this study are summarised in Table 1.

**TABLE 1:** Themes and sub-themes.

Themes	Sub-themes
Teamwork between registered nurses and medical doctors	Continuous communication between registered nurses and medical doctors Improvement of positive attitude between medical doctors and registered nurses Management and peer support
Conducting different types of staff development programmes	Peer seminars Workshops Provision of in-service training
Appropriate and adequate resources within the NICU	Purchasing appropriate equipment Proper inventory of available equipment Employment of qualified and experienced registered nurse

NICU, neonatal intensive care units.

### Theme 1: Teamwork between registered nurses and medical doctors

The teamwork between registered nurses and medical doctors can be successful only when both are willing to listen, support each other, and respond to questions and comments appropriately to maintain a professional work environment when caring for neonates. Registered nurses’ work-related support needs for teamwork resulted in three sub-themes, namely continuous communication between registered nurses and medical doctors, occupational and social support, and improvement of positive attitude between medical doctors and registered nurses.

#### Sub-theme 1.1: Continuous communication between registered nurses and medical doctors

The participants described that there is a need for continuous communication between registered nurses and medical doctors to provide quality care for neonates and as a form of promoting teamwork. This sub-theme was supported by two participants and indicated that:

‘There is a need for continuous communication between registered nurses and medical doctors to provide quality care for neonates and when they’re this kind of communication between nurses and doctors will promote teamwork amongst them.’ (Participant 1)

‘Continuous communication between registered nurses and medical doctors is necessary to convey the information that is needed to care for neonates.’ (Participant 2)

#### Sub-theme 1.2: Improvement of positive attitude between medical doctors and registered nurses

Registered nurses indicated that a positive attitude between medical doctors and registered nurses would help to promote a good working relationship amongst them, and consequently improve the quality of care that should be given to neonates. A positive attitude enables healthcare professionals to work together as colleagues, remain hopeful, and see the best even in difficult situations. Registered nurses ascertained that:

‘Nurses and doctors need to improve their attitudes towards each other to promote a good working relationship amongst each other.’ (Participant 1)

‘Nurses and doctors need to work on the negative attitude that they have amongst each other because a positive attitude is the most important thing needed to provide quality care for neonates that are admitted in NICU.’ (Participant 6)

‘Managers need to assist nurses and doctors to work on their attitudes so that patients can be able to receive holistic medical care from both the nurses and doctors.’ (Participant 5)

#### Sub-theme 1.3: Management and peer support

Management and peer support was regarded as one of the work-related supports needed by registered nurses working in a specific NICU to cope with work-related stressors. During unstructured individual interviews, registered nurses indicated the following:

‘I have the desired need of receiving support from management because we are short-staffed and NICU is stressful. Also as colleagues, we need to support one another especially when someone is having personal and work-related problems. This can be done by asking another registered nurse to work on your behalf on that day so that can attend to your problems and go back to work on the following day if you have to continue with the shift.’ (Participant 3)

‘We have to become friends with our colleagues and when we are friends it’s easy to talk about work and home-related problems. Therefore, colleagues must support one another with work and personal related problems because it will assist them to cope with work-related issues.’ (Participant 8)

### Theme 2: Conduct different staff development programmes

The second theme that emerged was conducting different types of staff development programmes for registered nurses working in the NICU. Registered nurses working in the NICU situated in Tshwane District, Gauteng Province must be given staff development programmes formally or informally to develop their skills, knowledge and competencies required to care for the admitted neonates. The staff development theme resulted in three sub-themes, namely peer seminars, workshops, and provision of in-service training.

#### Sub-theme 2.1: Peer seminars

During interviews, participants indicated that there is a need for work-related peer seminars where nurses can teach and learn from one another. The peer seminars will assist them to gain the knowledge needed in the provision of care to neonates who are admitted to NICU. In support of this, two participants indicated that:

‘Peer seminars must be conducted by qualified and experienced registered nurses and doctors to share the knowledge with junior staff on how to provide quality care for neonates that are admitted in NICU.’ (Participant 3)

‘I am a junior registered nurse, and I would like my senior colleagues to support us by providing peer seminars to teach us how to manage neonates because it is challenging to work in NICU.’ (Participant 4)

#### Sub-theme 2.2: Workshops

Registered nurses indicated the need for work-related workshops as one of the strategies for staff development. Workshops as documented by participants have an important role in influencing quality patient care. During interviews, registered nurses indicated the following:

‘We need to attend workshops where professionals can share knowledge on how to care for the neonates in our unit.’ (Participant 2)

‘There is a need of having continuous workshops that can be used to teach and learn from one another and the person who is teaching on that day should come prepared and others must come ready to learn.’ (Participant 9)

‘Workshops are needed to equip nurses about effective management styles that must be used to care for neonates and to reduce potential complications that are likely to arise in the units.’ (Participant 1)

#### Sub-theme 2.3: Provision of in-service training

Participants mentioned in-service training as one of the staff development work-related support needs that can be used to capacitate them on how to use new equipment and gain knowledge about best practices for providing quality care for neonates. As directly quoted, one of the participant said:

‘NICU staff should receive in-service training when the hospital is purchasing new equipment because we will find it easier to use the equipment and provide quality care.’ (Participant 3)

Furthermore, the need for in-service training is needed by less experienced registered nurses in the NICU and by those who do not have specialisation in ICU to improve their knowledge and skills in providing care to neonates. The direct quotation from the participants to support this sub-theme is as follows:

‘There is a need for in-service training for registered nurses that have less than five years of working in NICU because they need the skill and knowledge of caring for neonates.’ (Participant 3)

‘There is a need for in-service training for registered nurses that do not have the clinical specialisation that is relevant to provide adequate quality care for neonates.’ (Participant 4)

### Theme 3: Adequate resources within the workplace

Availability of adequate resources (both material and human) within the workplace was verbalised as one of the work-related support needs for registered nurses working in the NICU. Resources play a significant role in providing nursing care to patients and this theme was justified by three sub-themes which are purchasing equipment that is needed in the unit, systematic inventory control of available equipment, and employment of qualified and experienced permanent staff.

#### Sub-theme 3.1: Purchasing equipment needed in the unit

Registered nurses indicated that they need new equipment to work more efficiently and provide quality care for neonates admitted to NICU. The consensus on purchasing new equipment will contribute to offering quality care to patients.

‘There is a need of purchasing new equipment in this NICU and the quality ones for that matter because we don’t have enough equipment to provide quality care for neonates.’ (Participant 9)

‘If they want us to provide quality care for neonates, they need to purchase new equipment.’ (Participant 4)

#### Sub-theme 3.2: Inventory control of available equipment

Participants indicated that there is a need for standardised protocol that must be followed by professional nurses to record and manage equipment in the NICU. Inventory control of equipment must be done in the NICU and every ward of the hospital to prepare for emergencies to empower nurses to provide care that is aimed at saving lives. Inventory control of equipment is captured from the participants in the following manner:

‘Asset management is needed to make sure that you have the correct and working equipment in cases of emergencies to save the life of neonates.’ (Participant 7)

‘There is a need of having standardised protocols that can be used by professional nurses for assert management of equipment and this will enable us to carry out our duties without any struggle in cases of emergencies.’ (Participant 9)

#### Sub-theme 3.3: Employment of qualified and experienced permanent registered nurses

Registered nurses indicated that there is a need to employ qualified permanent staff to provide quality care for neonates. Also, there are patients admitted to NICU who present with complex neonatal conditions and require specialised care. Registered nurses who participated in this study indicated the following:

‘There is a need of employing well-trained registered nurses to provide quality care for neonates other than depending on agencies because sometimes they will give you a professional nurse that is not knowledgeable about NICU.’ (Participant 7)

‘NICU is not like any other wards, we are nursing fragile patients so there is a need of employing experienced and specialised nurses to provide quality care for neonatal care.’ (Participant 2)

## Discussion

Participants in this study indicated that improved teamwork between registered nurses and medical doctors is one of the work-related needs that must be practised to improve nurses’ well-being. According to participants, functional work-related teams would support the registered nurses’ needs and improve the working relationship between them and doctors. The findings are supported by McFarland, Shen and Holcombe (2017:121) when they asserted that effective communication between medical staff is helpful to improve positive patient outcomes. Therefore, this can be achieved by providing in-service training on effective communication as it will help to prevent many blunders within the NICU which are avoidable (Manojlovich 2010:941; Park, Park & Yu 2018:166).

The findings of this study also revealed that there is a need for better working relationship between registered nurses and doctors. It was indicated that colleagues need to improve their attitudes toward each other, and this must be addressed as a matter of urgency, and ensure that quality care is rendered in the NICU. Tang et al. (2013:291) indicated that nurses and doctors have a negative attitude towards each other, and this is affecting the provision of quality care for patients. Pakpour, Ghafourifard and Salimi (2019:111) concurred that nurses and doctors should foster a new culture of working as a team with the common aim of providing quality care to patients.

Management and peer support was also regarded as one of the work-related supports that is needed by registered nurses working in the NICU. In support of this finding, Gutierrez et al. (2012:1613) asserted that registered nurses receiving a high-level support from their co-workers and their managers have low levels of job stress. Therefore, there is a need for management to develop interventions that can be used to educate registered nurses about the benefits of supporting one another. Also, managers should be supportive of registered nurses to prevent work-related stress, as in most cases nurses are understaffed and work under difficult conditions.

The findings of this study together with previous findings from India revealed that capacitating registered nurses working in NICU should be mandatory as it increases their knowledge and prevents possible mistakes which may lead to litigations (Kalyan & Vatsa 2014:120). Evans et al. (2018:207) stated that there is an urgent need for using peer-assisted learning to improve and maintain the intrapartum skills of healthcare workers in sub-Saharan Africa. Peer learning in Uganda has shown that this is feasible and it can be done using simulation-based training (Evans et al. 2018:207). Evans et al. (2018) also revealed that peer seminars in India have shown the potential of increasing the competency of junior staff members. Mentors and experienced staff members assist in developing competencies needed in delivering safe patient care that meets established standards of practice (Lin, Viscardi & McHugh 2014:439).

The results of this study also indicated that nurses have the desired need for attending workshops as it will keep them abreast with the latest developments within the field, i.e. it was indicated that nurses who had not attended pain management workshops in the previous 2 years have shown less knowledge of neonatal pain treatment (Tarjoman et al. 2019:137). However, after attending the workshop, they showed significant improvement in their knowledge of pain management (Tarjoman et al. 2019:137). Twamley et al. (2013:213) concurred that workshops have a significant role in improving the knowledge of providing palliative care. Therefore, workshops should be adopted as one of the support measures that can be used to capacitate nurses on how to manage and handle unforeseen circumstances that may occur in the NICU.

Participants indicated the need for in-service training to support novice registered nurses. This is supported by Kalyan and Vatsa (2014:120) as they indicated that there is a need to provide in-service training to novice registered nurses to improve the quality of care for patients. Chaghari et al. (2017:26) stated that in-service training is an unavoidable need to enhance the quality of inpatient treatment. Empowering nurses with practical knowledge will assist them in completing occupational tasks and attaining higher mastery of professional skills (Chaghari et al. 2017:26). Hence, there is a need to provide in-service training for non-specialised and less experienced registered nurses working in the NICU.

Phillips et al. (2012:31) posited that despite the development of tools and resources to assist hospitals in emergency preparedness, there appears to be a lack of adequate resources to the adverse of the hospital and staff. Infants in NICU are a vulnerable group, and their care must be considered during the emergency preparedness process. Infants pose special problems because of their unique and complicated physiological needs (Phillips et al. 2012:31). Neonatal intensive care unit patients are some of the most vulnerable within the hospital, as such emergency preparedness for this group should be a top concern. However, fewer resources are an indication of poor emergency planning (Phillips et al. 2012:31). The authors designed a checklist that can be used by NICU emergency subcommittees to monitor equipment for functionality and those that are due for service. This checklist can be refined according to country specifics and needs.

Work-related support needs linked to the availability of resources are documented in this study. Registered nurses indicated that management must purchase equipment that will help them provide quality care for neonates in NICU. Staebler (2013:447) stated that there is a need to involve frontline workers when purchasing the equipment to avoid purchasing the equipment that the front liners do not need and/or have knowledge of. More research is needed on how nurses can be empowered to take manage equipment in their wards.

The results of this study concur with Mirlashari et al. (2016:317), that neonatal care given in NICU must be under the supervision of qualified specialist registered nurses. Also, registered nurses who are working in NICU must have a minimum of five years of work experience in neonatal medical, surgical or operating rooms (Mirlashari et al. 2016:317). Mirlashari et al. (2016) go further to indicate that due to the staffing deficit, this is not always attainable. Mekonnen and Desalegn (2018:412) stated that the NICU in Ethiopian hospitals are understaffed and are exposing new-borns to a variety of problems that increase their chances of non-survival. This is an indication that there is a need to equip the NICU with experienced nurses who can provide quality care to neonates and prevent complications that may result in deaths.

### Strength and limitations

The findings of this study have revealed that work-related support needs for registered nurses working in NICU will improve their well-being and working relationships with other team members, and consequently deliver quality care for neonates. The limitation of the study is that it only focused on registered nurses and did not consider other team members working in the selected NICU in the hospital. Also, the study was conducted in only one academic hospital in the Tshwane District. However, similar studies can be conducted in other districts and other provinces of SA. The other provinces may use the findings of this study to offer work-related support needed by registered nurses working in their NICU.

### Recommendations

The findings of this study recommend the development of strategies that can be used to support the work-related needs of registered nurses working in public hospitals to ensure that they are well taken care of. This study also recommends the adoption of these work-related support needs of registered nurses working in the NICU both in undergraduate and postgraduate curricula of NICU-related courses. The results of this study also recommend the adoption of these work-related support needs for hospital management to use them to assist registered nurses working in the NICU. Furthermore, this study recommends that registered nurses working in NICU must consider using this work-related support as and when the need arises.

## Conclusion

The current study explored and described the work-related support needs of registered nurses working in the NICU of an academic hospital in a specific district of Tshwane in the Gauteng province of SA. The study resulted in three themes which are teamwork between registered nurses and medical doctors, staff development programmes for nurses, and appropriate and adequate resources for NICU. These findings point to the development of strategies that can be used to meet the identified work-related support needs of registered nurses working in the NICU, thus improving their well-being and consequently assisting them to provide high-quality care to neonates.
